# Supplementary material for: The Role of EEG in the Diagnosis, Prognosis and Clinical Correlations of Dementia with Lewy Bodies—A Systematic Review
Source: Diagnostics (Basel). 2020 Aug 20;10(9):616. doi: 10.3390/diagnostics10090616 (PMC7555753; doi:10.3390/diagnostics10090616)
Supplement: Supplementary file 1 [file diagnostics-10-00616-s001.zip › Supplemental file-1 tables S1-S6 Diagnostics.pdf]

Table S1. PRISMA-DTA checklist.

EMBASE search strategy 6th August 2020
1. DLB.mp.
2. lewy* bod*.mp.
3. exp Lewy body/
4. exp Dementia with Lewy Bodies/
5. LBD.mp.
6. 1 or 2 or 3 or 4 or 5
7. exp Electroencephalography/
8. (EEG or electroencephalogra*).mp.
9. compressed spectral arrays.mp.
10. compress* spectr* array*.mp.
11. (grand total EEG or grand total electroencephalogr*or GTE).mp.
12. (modified grand total EEG or modified grand total electroencephalogr*or modified GTE).mp.
13. (frontal intermittent rhythmic delta activit* or FIRDA*).mp.
14. spectr* power*.mp.
15. (EEG coherence or electroencephalogra* coherence).mp.
16. (EEG microstate* or electroencephalogra* microstate*).mp.
17. (eLORETA or LORETA or low resolution brain electromagnetic tomography).mp.
18. Lagged linear connectivity.mp.
19. phase lag index.mp.
20. phase transfer entropy.mp.
21. network connectivity*.mp.
22. (alpha rhythm or posterior dominant rhythm or posterior basic rhythm or alpha frequenc*).mp.
23. (slow activit* or slowing or transient slow* or slow wave* or slow-wave*).mp.
24. (theta or delta or alpha1 or pre-alpha).mp.
25. 7 or 8 or 9 or 10 or 11 or 12 or 13 or 14 or 15 or 16 or 17 or 18 or 19 or 20 or 21 or 22 or 23 or
26. 6 and 25
MEDLINE search strategy 6th August 2020
1. (DLB or LBD).mp.
2. Lewy Body Disease/
3. (lewy* adj2 bod*).mp.
4. Dement* with Lewy Bod*.mp.
5. 1 or 2 or 3 or 4
6. exp Electroencephalography/
7. (EEG or electroencephalogra*).mp.
8. compressed spectral arrays.mp.
9. compress* spectr* array*.mp.
10. (grand total EEG or grand total electroencephalogr*or GTE).mp.
11. (modified grand total EEG or modified grand total electroencephalogr*or modified GTE).mp.
12. (frontal intermittent rhythmic delta activit* or FIRDA*).mp.
13. (alpha rhythm or posterior dominant rhythm or posterior basic rhythm or alpha frequenc*).mp.
14. (slow activit* or slowing or transient slow* or slow wave* or slow-wave*).mp.
15. (theta or delta or alpha1 or pre-alpha).mp.
16. spectr* power*.mp.
17. (EEG coherence or electroencephalogra* coherence).mp.
18. (EEG microstate* or electroencephalogra* microstate*).mp.
19. (eLORETA or LORETA or low resolution brain electromagnetic tomography).mp.
20. lagged linear connectivity.mp.
21. phase lag index.mp.
22. phase transfer entropy.mp.

23. network connectivity*.mp.
24. 6 or 7 or 8 or 9 or 10 or 11 or 12 or 13 or 14 or 15 or 16 or 17 or 18 or 19 or 20 or 21 or 22 or 23
25. 5 and 24

PsycINFO search strategy 6th August 2020

1. (DLB or LBD).mp.
2. Dement* with Lewy Bod*.mp.
3. lewy* bod*.mp.
4. exp Lewy body/
5. exp Dementia with Lewy Bodies/
6. 1 or 2 or 3 or 4 or 5
7. exp Electroencephalography/
8. (EEG or electroencephalogra*).mp.
9. compress* spectr* array*.mp.
10. (grand total EEG or grand total electroencephalogr*or GTE).mp.
11. (frontal intermittent rhythmic delta activit* or FIRDA*).mp.
12. (alpha rhythm or posterior dominant rhythm or posterior basic rhythm or alpha frequenc*).mp.
13. (slow activit* or slowing or transient slow* or slow wave* or slow-wave*).mp.
14. (theta or delta or alpha1 or pre-alpha).mp.
15. spectr* power*.mp.
16. EEG coherence.mp.
17. EEG microstate*.mp.
18. eLORETA.mp.
19. lagged linear connectivity.mp.
20. phase lag index.mp.
21. phase transfer entropy.mp.
22. network connectivity*.mp.
23. 7 or 8 or 9 or 10 or 11 or 12 or 13 or 14 or 15 or 16 or 17 or 18 or 19 or 20 or 21 or 22
24. 6 and 23

Table S2. EEG acquisition

	Study	EEG Leads Placement System	Number of EEG Electrodes/ Channels	Duration of Recording (min)	Machine	Sampling Frequency (Hz)	Frequency Filter	Described Preprocessing	Analysis Tool	Frequency Band Definition (hz)
1	Briel 1999 [25]	10-20	20 electrodes	NA	Galileo VEGA 24w system.	1000	70	No	visual	NK
2	Roks 2008 [26]	10-20	NA	NA	OSG digital equipment (Brainlab; OSG b.v., Rumst, Belgium)	NA	NA	No	visual	NK
3	Lee 2015 [27]	10-20	21 channels	30	OSG digital equipment (Brainlab; OSG b.v., Rumst, Belgium)	NA	0.27–70 Hz,	No	visual	NK
4	Barber 2000 [28]	NA	24 channels	NA	Walter-Graphtek digital signal machine	NA	NA	No	visual	NK
5	Londos 2003 [29]	10-20	16 or 24 electrodes	NA	Siemens Elema or Nihon Kohden	NA	Time constant 0.3s; low pass filter 70 Hz	No	visual	NR
6	Walker 2000b [30]	10-20	17 electrodes	60	NEUROSCAN SynAmps System	NA	NA	Yes	NA	δ=0.5-3.9
7	Walker 2000a [31]	10-20	17 electrodes	NA	NEUROSCAN SynAmps System	NA	NA	No	NA	NK
8	Kai 2005 [32]	10-20	14 electrodes	NA	NEC electroencephalograph SYNAFIT 1000	1000	1000-Hz cutoff and 0.1-s time constant	Yes	-	ß=13.1-19.9; α=7.8-13.1; θ=3.9-7.8; δ=0.5-3.9
9	Andersson 2008 [33]	10-20	19 electrodes	NA	Nervus (Viasys Healthcare Inc., Madison, Wisc., USA	256	high-pass filter at 0.16 Hz and a low-pass filter at 500 Hz.	Yes	Nervus Reader 3.4, Viasys Healthcare	ß=13-32; α=8-13; θ=4-8; δ=0.5-4
10	Bonnani 2008 [34]	10-20	21 electrodes	10	NEUROSCAN SynAmps System	1024	0.5-70 Hz, notch	Yes	NEUROSCAN SynAmps System	α=8-12; pre α=5.6-7.9; θ=4-5.5; δ=1-3.9
11	Bonnani 2010 [35]	10-20	21 channels	30	NEUROSCAN SynAmps System	2000	0.15 Hz and 100 Hz,	Yes	NEUROSCAN SynAmps System	α=8-12; pre α=5.6-7.9; θ=4-5.5; δ=1-3.9
12	Bonnani 2015 [36]	10-20	21 channels	30	NEUROSCAN SynAmps System	1024	0.5-70 Hz	Yes	NEUROSCAN SynAmps System	α=8-12; pre α=5.6-7.9; θ=4-5.5; δ=1-3.9

13	Bonnani 2016 [37]	10-20; or 10-5	19+2 electrodes; 128 electrodes	NA	NA	NA	NA	Yes	NA	α=8-12;<br>pre α=6-7.5;<br>θ=4.5-5.5;<br>δ=3-4
14	Stylianou 2018 [38]	10-5	128 electrodes	NA	ASA-Lab software (ANT Neuro, Netherlands	1024	4 Hz high-pass and a 46 Hz low-pass filter	Yes	MATLAB 8.5 (MathWorks)	ß=14-20.75; α=8-13.75;<br>θ=4-7.75;
15	Snaedal 2012 [39]	10-20	19 electrodes	3	NicoletOne EEG Systems (CareFusion)	NA	NA	Yes	Matlab (MathWorks)	γ=25-40<br>ß$_2$=17.5-25<br>ß$_1$=12.5-17.5;<br>α$_2$=9.5-12.5;<br>α$_1$=7.5-9.5<br>θ=3.5-7.5;<br>δ=0.5-3.5
16	Franciotti 2013 [40]	10-20	19 electrodes	30	NA	NA	NA	Yes	NA	α=8-12.5;<br>pre α=5.5-8; θ=4-5.5;<br>δ=1-4
17	Garn 2017 [41]	10-20	NA	NA	Sienna digital EEG (EMS Biomedical, Austria) & a-EEG & Neurospeed software (Alpha Trace, Austria)	256	0.3-70	Yes	Matlab R2013b	ß=13-20; α=8-13;<br>θ=4-8;<br>δ=2-4
18	Engedal 2015 [42]	10-20	19 electrodes	≥3	NicoletOne EEG Systems (Natus ®)	1,024, 1,000, 512, 500, 256, 250 or 200 Hz in the 6 centers.	NA	No	MATLAB environment (MathWorks)	γ=25-40<br>ß$_2$=17.5-25<br>ß$_1$=12.5-17.5;<br>α$_2$=9.5-12.5;<br>α$_1$=7.5-9.5<br>θ=3.5-7.5;<br>δ=0.5-3.5
19	Ferreira 2016 [43]	10-20	19 electrodes	≥3	NicoletOne EEG Systems (Natus ®)	NA	NA	No	MATLAB environment (MathWorks)	γ=25-40<br>ß$_2$=17.5-25<br>ß$_1$=12.5-17.5;<br>α$_2$=9.5-12.5;<br>α$_1$=7.5-9.5<br>θ=3.5-7.5;<br>δ=0.5-3.5
20	Colloby 2016 [44]	10-5	128 electrodes	2.5	ASA-Lab software (ANT Neuro, Netherlands	1024	NA	No		γ=25-40<br>ß$_2$=17.5-25<br>ß$_1$=12.5-17.5;<br>α$_2$=9.5-12.5;<br>α$_1$=7.5-9.5<br>θ=3.5-7.5;

										δ=0.5-3.5
21	van Dellen 2015 [45]	10-20	21-channels	NA	Brainlab; OSG b.v., Rumst, Belgium	500	0.16-70	Yes	Brain Wave	ß=13-30; α=8-13; θ=4-8; δ=0.5-4
22	Peraza 2018 [46]	10-5	128 electrodes	NA	ASA-Lab software (ANT Neuro, Netherlands	1024	0.3 Hz and 54 Hz	Yes	EEGLAB MATLAB functions (R2012; MathWorks)	ß=13-30; α=8-13; pre α=5.5-8; θ=4-5.5; δ=0.5-4
23	Dauwan 2016b [47]	10-20	21 channels	20	Brainlab; OSG b.v., Rumst, Belgium	500	time constant of 1 s; low pass filter 70 Hz	Yes	BrainWave software	ß=13-30; α=8-13; θ=4-8
24	Dauwan 2016a [48]	10-20	21 channels	20	Brainlab; OSG b.v., Rumst, Belgium	500	time constant of 1 s; low pass filter 70 Hz	Yes	BrainWave software	ß=13-30; $α_2$=10-13; $α_1$=8-10; θ=4-8; δ=0-4
25	Dauwan 2018 [49]	10-20	21 channels	20	Brainlab; OSG b.v., Rumst, Belgium	500	time constant of 1 s; low pass filter 70 Hz	Yes	BrainWave software	ß=13-30; $α_2$=10-13; $α_1$=8-10; θ=4-8; δ=0-4
26	van der Zande 2018 [50]	10-20	21 electrodes	20	Brainlab; OSG b.v., Rumst, Belgium	500	time constant of 1 s; low pass filter 70 Hz	Yes	MATLAB 2011a (MathWorks)	γ=30-48 ß=13-30; $α_2$=10-13; $α_1$=8-10; θ=4-8; δ=0-4
27	Babiloni 2017 [51]	10-20	19+2 electrodes	≥5	NA	≥128	0.01 to 100	Yes	MATLAB 2010b (Mathworks)	IAF and TF
28	Babiloni 2018a [52]	10-20	19+2 electrodes	5	Multiple systems	≥128	0.01 to 100	Yes	eLORETA	IAF and TF
29	Babiloni 2018b [53]	10-20	19 electrodes	≥5	NA	≥128	0.01 to 100	Yes	Matlab 6.5 (Mathworks) and eLORETA	IAF and TF
30	Babiloni 2019 [54]	10-20	19 electrodes	5	NA	≥128	0.01-100 hz	Yes	eLORETA	IAF and TF
31	Schumacher 2019 [55]	10-5	128 electrodes	NA	ASA-Lab software (ANT Neuro, Netherlands	1024	0.3 Hz and 54 Hz	Yes	MATLAB R2017a	ß=13-30; α=8-13; high θ=5.5-8; θ=4-5.5; δ=0.5-4
32	Tanaka 2017 [56]	10-20	21 or 19 electrodes	NA	NA	200	NA	No	NA	NR
33	Liedorp 2009 [57]	10-20	21 channels	30	Nihon Kohden digital EEG apparatus (EEG 2100), OSG digital equipment (Brainlab)	200 for Nihon Kohden and 500 for OSG	time constant of 1 s; low pass filter 70 Hz	No	Visual	NR
34	Kurita 2010 [58]	Fz, Cz, Pz, and Oz	4 electrodes	NA	Neuropack Ramda (Nihon Kohden, Tokyo, Japan)	NA	NA	No	NA	NR

35	Perriol 2005 [59]	10-20	NA	NA	SYNAMP amplifiers and the SCAN v3.0 software (Neuroscan Inc., USA). STIM package's Gentask module (Neuroscan Inc., USA).	2000	1–100 Hz	No	NA	NR
36	Pugnetti 2010 [60]	10-20	30 electrodes	2	Compumedics Neuroscan Synamps, El Paso, Texas, USA	500	1-40;	Yes	NA	ß$_3$=25.5-35 ß$_2$=15.5-25 ß$_1$=13-15.5; α_2=11-13; α_1=8-11; θ=4.5-7.5; δ=1-4
37	Mehraram 2019 [61]	10-5	128 electrodes	2.5	ASA-Lab software (ANT Neuro, Netherlands	1024	0.5 Hz and 80 Hz	Yes	MATLAB 9.2 2017	ß=14-20.5; α=8-13.5; θ=4-7.5
38	Aoki 2019 [62]	10-20	19 channels	20	EEG-1000/EEG-1200; Nihon Kohden Inc., Tokyo, Japan)	500	0.53–120 Hz	Yes	Neuroworkbench software (Nihon Kohden Inc.) and eLORETA-ICA	γ=30-60 ß=13-30; α=8-13; θ=4-8; δ=2-4
39	Schumacher 2020a [63]	10-5	128 electrodes	5	ANT Neuro, The Netherlands	1024	0.3-54	Yes	NA	IAF α=8-12 Hz
40	Franciotti 2020 [64]	10-20 or 10-5	19 or 128 electrodes	30	NA	NA	NA	Yes	NA	α=8-12 Hz pre- α=6-7.5 Hz θ=4-5.5 Hz ∂=3-3.5 Hz
41	Massa 2020 [65]	10-20	21 electrodes	20	NA	NA	NA	Yes	NA	α=8-12 Hz θ=4-8 Hz
42	van der Zande 2020 [66]	10-20	21 electrodes	20	Brainlab; OSG b.v., Rumst, Belgium	500	1-70	Yes	BrainWave software,	ß=13-30; α_2=10-13; α_1=8-10; θ=4-8; δ=0.5-4
43	Schumacher 2020b [67]	10-5	128 electrodes	5	ANT Neuro, The Netherlands	1024	0.3-54	Yes	MATLAB R2017a	ß=13-30; α=8-13; pre- α=5.5-8; θ=4-5.5; δ=2-4

Table S3. Potential confounders of EEG analyses

	Study	Participants Alerted when Drowsy?	Artefact Monitored (with EOG, ECG etc)	Artefact Excluded	Medication	Proportion on Medication Matched between Groups	Age-Matched	Cognitive Score-Matched	Blinding of Assessor/Technician to Diagnosis
1	Briel 1999 [25]	NK	NK	NR	Not known	Not known	Yes	Yes-CDR	Yes
2	Roks 2008 [26]	Yes	NK	NR	Antipsychotic, benzodiazepine, CHEIs	No (DLB>)	Yes	Yes-DLB vs AD, not HC	Yes
3	Lee 2015 [27]	Yes	NK	NR	Antipsychotic, benzodiazepine, CHEIs, antidepressant	No (DLB>)	Yes	Yes	Yes
4	Barber 2000 [28]	Yes	NK	NR	Not known	Not known	No	No	Yes
5	Londos 2003 [29]	Yes	NK	NR	Not known	Not known	Yes	NK	Yes
6	Walker 2000b [30]	NK	Yes	Yes	Not known	Not known	Yes	Yes-DLB vs AD, not HC	NK
7	Walker 2000a [31]	NK	Yes	NA	Not known	Not known	Yes	Yes-DLB vs AD vs Vad, not HC	Yes
8	Kai 2005 [32]	NK	NK	Yes	L-dopa, amantadine, dopamine agonist	No (DLB>)	Yes	Yes	NK
9	Andersson 2008 [33]	Yes	Yes	Yes	Antipsychotic, benzodiazepine, antidepressant	No (DLB>)	Yes	Yes-DLB vs AD, not HC	NK
10	Bonnani 2008 [34]	Yes	Yes	Yes	Excluded antidepressants, anticonvulsants, benzodiazepines,antipsychotics, or anticholinergic	NR	Yes	Yes-DLB vs AD vs PDD, not HC	Yes
11	Bonnani 2010 [35]	Yes	Yes	Yes	NK	Not known	Yes	Yes-DLB vs AD, not HC	Yes
12	Bonnani 2015 [36]	Yes	Yes	Yes	Excluded antidepressants, anticonvulsants, benzodiazepines	NR	Yes	Yes MCI-DLB vs MCI AD; DLB vs AD; Not MCI vs HC/DLB/AD	Yes
13	Bonnani 2016 [37]	NK	NK	Yes	NK	Not known	No	Yes	Yes
14	Stylianou 2018 [38]	NK	NK	Yes	L-dopa (stop at 1-3 hours before), CHEi	No (LBD>)	Yes	Yes	NK

					(stop at least 4 hours before)				
15	Snaedal 2012 [39]	Yes	Yes-EOG and ECG	Yes	NK	Not known	No	NK	NK
16	Franciotti 2013 [40]	NK	NK	Yes	NK	Not known	Yes	Yes-DLB vs AD, not HC	NK
17	Garn 2017 [41]	NK	Yes	Yes	NK	Not known	No	No	NK
18	Engedal 2015 [42]	Yes	Yes	NA	Antidepressant, antipsychotic, hypnotics, ChEI, memantine, painkillers	Unclear	NK	NK	Yes
19	Ferreira 2016 [43]	Yes	Yes	NA	Excluded psychotropic drugs	NR	No	Yes-DLB vs AD, not HC/MCI	NK
20	Colloby 2016 [44]	NK	NK	NA	NK	Not known	Yes	Yes-DLB vs AD, not HC	NK
21	van Dellen 2015 [45]	Yes	NK	Yes	Antipsychotic, ChEI, AED	No (DLB>)	Yes	No	No
22	Peraza 2018 [46]	Yes	NK	Yes	Dopaminergic, ChEI	No (LBD>)	Yes	Yes-DLB vs PDD vs AD, not HC	Yes
23	Dauwan 2016b [47]	NK	NK	Yes	Antipsychotic, ChEI, AED	No (DLB>)	Yes	No	NK
24	Dauwan 2016a [48]	Yes	NK	Yes	Antipsychotic, ChEI, AED	No (DLB>)	Yes	No	NK
25	Dauwan 2018 [49]	Yes	NK	Yes	Hallucination-associated drugs	Unclear	Yes	Yes	NK
26	van der Zande 2018 [50]	Yes	NK	Yes	ChEI, benzodiazepine, antidepressant, antipsychotic	No (DLB>)	Yes	No	NK
27	Babiloni 2017 [51]	Yes	Yes	Yes	ChEI, memantine, benzodiazepine, antidepressant; Stopped benzodiazepine and antidepressant for 24h	Unclear	Yes	Yes-DLB vs PDD vs AD, not HC	Yes
28	Babiloni 2018a [52]	NK	Yes	Yes	ChEI, memantine, benzodiazepine, antidepressant,	Unclear	Yes	Yes-DLB vs PDD vs AD, not HC	NK
29	Babiloni 2018b [53]	Yes	Yes	Yes	ChEI, antidepressant, antipsychotic, anxiolytic	Unclear	Yes	Yes-DLBMCI vs ADMCI, not HC	Yes

30	Babiloni 2019 [54]	NK	Yes	Yes	Exclude anticholinergic, antipsychotic, analgesics, sedative/hypnotic, antiparkinson drug	NR	Yes	Yes-DLBMCI vs ADMCI, not HC	Yes
31	Schumacher 2019 [55]	Yes	NK	Yes	PD med, ChEI	No (LBD>)	Yes	No	Yes
32	Tanaka 2017 [56]	NK	NK	NA	NK	Not known	No	No	NK
33	Liedorp 2009 [57]	Yes	NK	NR	NK	Not known	NK	NK	NK
34	Kurita 2010 [58]	NK	NK	NA	Excluded antipsychotic	NR	Yes	Yes-DLB vs PDD vs AD, not HC	NK
35	Perriol 2005 [59]	NK	Yes	NA	L-dopa, ChEI	No (LBD>)	Yes	Yes	NK
36	Pugnetti 2010 [60]	Yes	Yes	Yes	L-dopa	No (PDD>DLB)	Yes	No	NK
37	Mehraram 2019 [61]	Yes	No	Yes	ChEI, doparminergic	Yes ChEI; no for doparminergic	Yes	No	NK
38	Aoki 2019 [62]	Yes	No	Yes	Excluded ChEI, L-dopa, DA agonists, and antipsychotics	NR	No	No vs HC	NK
39	Schumacher 2020a [63]	Yes	No	Yes	ChEI and PD meds	Yes ChEI; no for doparminergic	Yes	Yes	Yes
40	Franciotti 2020 [64]	Yes	Yes	Yes	ChEI, L-dopa, antidepressant, antipsychotic, benzodiazepine	Yes ChEI and antidepressant, no for antipsychotic, benzodiazepine, L-dopa	Yes	Yes, No vs HC	NK
41	Massa 2020 [65]	Yes	Yes	Yes	Excluded cholinergic, antiepileptics, benzodiazepines and antipsychotics	NR	Yes	Yes, No vs HC and PD	NK
42	van der Zande 2020 [66]	Yes	No	Yes	ChEI, antidepressant, antipsychotic, benzodiazepine, dopaminergic, antiepileptic	Yes	Yes	Yes, no vs HC	Yes
43	Schumacher 2020b [67]	Yes	No	Yes	ChEI, PD meds	No	Yes	Yes, no vs HC	Yes

ChEI=cholinesterase inhibitors; NK=not known; NR=not relevant

Table S4. Quality of included studies

	Study	JBI Study checklist (/10)	EEG Checklist (/7)	Total (/17)	Overall Quality
1	Briel 1999 [25]	7	3	10	moderate
2	Roks 2008 [26]	9	6	15	high
3	Lee 2015 [27]	8	4	12	moderate
4	Barber 2000 [28]	8	3	11	moderate
5	Londos 2003 [29]	5	3	8	low
6	Walker 2000b [30]	7	5	12	moderate
7	Walker 2000a [31]	8	4	12	moderate
8	Kai 2005 [32]	6	5	11	moderate
9	Andersson 2008 [33]	7	5	12	moderate
10	Bonnani 2008 [34]	8	7	15	high
11	Bonnani 2010 [35]	6	6	12	moderate
12	Bonnani 2015 [36]	7	7	14	high
13	Bonnani 2016 [37]	7	4	11	moderate
14	Stylianou 2018 [38]	6	5	11	moderate
15	Snaedal 2012 [39]	6	1	7	low
16	Franciotti 2013 [40]	8	2	10	moderate
17	Garn 2017 [41]	8	3	11	moderate
18	Engedal 2015 [42]	7	3	10	moderate
19	Ferreira 2016 [43]	7	3	10	moderate
20	Colloby 2016 [44]	6	3	9	low
21	van Dellen 2015 [45]	7	4	11	moderate
22	Peraza 2018 [46]	7	5	12	moderate
23	Dauwan 2016b [47]	7	4	11	moderate
24	Dauwan 2016a [48]	7	3	10	moderate
25	Dauwan 2018 [49]	7	5	12	moderate
26	van der Zande 2018 [50]	7	4	11	moderate
27	Babiloni 2017 [51]	7	6	13	moderate
28	Babiloni 2018a [52]	7	5	12	moderate
29	Babiloni 2018b [53]	5	6	11	moderate
30	Babiloni 2019 [54]	6	7	13	moderate
31	Schumacher 2019 [55]	6	4	10	moderate
32	Tanaka 2017 [56]	5	1	6	low
33	Liedorp 2009 [57]	8	2	10	moderate
34	Kurita 2010 [58]	6	6	12	moderate
35	Perriol 2005 [59]	6	5	11	moderate
36	Pugnetti 2010 [60]	6	4	10	moderate
37	Mehraram 2019 [61]	6	4	10	moderate
38	Aoki 2019 [62]	8	4	12	moderate
39	Schumacher 2020a [63]	7	5	12	moderate
40	Franciotti 2020 [64]	6	5	11	moderate
41	Massa 2020 [65]	6	5	11	moderate
42	van der Zande 2020 [66]	7	7	14	high
43	Schumacher 2020b [67]	7	6	13	moderate

*overall score <10=low quality; 10-13=moderate quality; 14-17=high quality

Table S5. EEG definitions

Parameters	Definitions
Spectral power analysis	
Dominant Frequency (DF)	The mean frequency where the maximum power was represented in the selected epochs
Dominant Frequency range	The range of dominant frequencies in the 90 epochs
Frequency prevalence	Percent of epochs where prevalence of a DF band was observed
Band inscription	The percent of epochs where a peak of frequency was identified with a total amplitude above the mean amplitude of random peaks (noise)
Frequency ratio	band powers of pre-alpha or alpha versus delta, theta, pre-alpha or alpha
DF variability	The variability of DF across the analysed epoch
CSA pattern	1. Stable alpha: DF≥8 Hz, FP alpha ≥60%, DFV of alpha <0.6 Hz, mean DFV of all epochs <1.6 Hz, Band Inscription of pre-alpha, theta or delta activities <30% of epochs; 1 plus: DF DF≥8 Hz, FP alpha ≥60%, DFV of alpha ≥1.5 Hz.<br>2. Unstable alpha+ pre-alpha: dominant alpha (≥8 Hz) in <50% of epochs, mean DFV >2 Hz, dominant pre-alpha or theta (<8 Hz) in ≥40% epochs (FP pre-alpha >40%, BI of pre-alpha-theta-delta 50%)<br>3. Stable pre- alpha: absence of alpha, stable pre-alpha (DF≤7.9 Hz), ≥70% epochs, DF range 5.6–7.9 Hz, DFV <1.0 Hz<br>4. Unstable pre- alpha+theta/delta: absence of alpha, dominant pre-alpha In <70% epochs, dominant theta or delta in ≥40% epochs, DFV >2.0 Hz<br>5. Unstable theta or delta: absence of alpha, absence of alpha/pre-alpha dominant activity in 2 subsequent epochs, DFV >4 Hz.
Power	$(\text{Amplitude})^2$ measured in μV^2
Relative power	Ratio of signal power within a frequency band to the total signal power from all band
eLORETA	Exact low-resolution brain electromagnetic topography
Individual alpha frequency peak (IAF)	Maximum power density peak in the alpha range (6–14 Hz).
Transition frequency (TF)	Transition frequency between the theta and the alpha bands defined as the minimum of the rsEEG power density between 3 and 8 Hz (between the delta and the alpha power peak).
Determination of individual frequency bands	Based on TF and IAF, the frequency band range for each subject were estimated as follows: delta from TF-4 Hz to TF -2 Hz, theta from TF-2 Hz to TF, low-frequency alpha band (alpha 1 and alpha 2) from TF to IAF, and high-frequency alpha band (or alpha 3) from IAF to IAF +2 Hz.
Connectivity-single connection estimation	
Coherence	A real function ranging from 0 to 1, indicating the amount of correlation between signals x and y as a function of the frequency. Values of high coherence, near 1.0, indicate a large degree of shared activity, while low values, near 0.0, denote a minimum of shared activity. Coh=[Sxy]2 / (Sxx_Sxy), where Sxy, Sxx, and Syy are the cross-spectrum and autospectrum estimates of leads x and y, respectively.
Phase Lag Index	Measure the regional relation among all electrode pairs. PLI uses the Hilbert transform to estimate consistent causal delay between two signal sources and it has been proven that this measure is less affected by the scalp's volume conduction, a problem with other measures such as spectral coherence or Pearson's correlation. PLI scores are bounded between 0 and 1, where 0 means lack of causal synchronisation and 1 full causal synchronisation.
Directed Phase transfer entropy	dPTE measures signals between 2 points and indicates which signal is a directional driver and which signal is a directional receiver of information, hence not just the strength but also the direction of connectivity.

Global field synchronisation	The percentage of EEG activities oscillating with a common phase at a given frequency irrespective of location; its measurement is based on several assumptions, notably that the amount of phase-locked oscillations detectable by an EEG recording is mediated through networks of cortico–cortical connections, high values indicating strong functional binding and low values indicating a state of more or less permanent disconnection.
Lagged linear connectivity	Linear measurements of the statistical interdependence of pairs of eLORETA cortical source activations estimated from scalp rsEEG rhythms at a given frequency.
Connectivity-whole network estimation	
Minimum Spanning Tree	A brain network is represented within the framework of graph theory by a graph comprised of nodes and edges; in EEG, the nodes are the electrodes and the edges are the connectivity between electrodes. Network connectivity can be estimated with the phase lag index (PLI), a connectivity measure resistant to the effects of the scalp's volume conduction or dPTE. The MST is then the network, which has the minimum number of strongest edges while connecting all nodes without cycling paths.
Betweenness centrality	Number of paths between all other nodes in the MST crossing the node of interest, divided by the total number of paths in the MST.
Degree	Number of edges connected to each node
Diameter	Longest distance between any 2 nodes in the MST network.
Eccentricity	Maximum distance between a node and any other node in the MST.
Leaf fraction	Ratio between number of leaf nodes (only one edge) divided by the total number of nodes within the MST.
Tree hierarchy	Th = leaf number/ (2m BC_{max}), in which m is the number of edges and BC_{max} is the highest betweenness centrality of any node in the tree. For a line-like topology, Th is equal to zero, for a star-like topology, Th =0.5, and for trees with a configuration between these two extreme situations, Th can have values of ≈1
EEG Microstates	Transient, patterned, quasi-stable EEG topographies that remain stable for a certain period of time (60–120 ms) before rapidly transitioning into a different topography that remains stable again.
Global field power (GFP)	The spatial standard deviation of the average-referenced signal across all electrodes and whose local maxima represent instants of highest field strength.
Microstate duration	The time during which all successive maps were assigned to the same microstate.
Microstate occurrence	Number of occurrences of each microstate per second
Microstate coverage	Percentage of total analysis time covered by each microstate
Event-related potentials	Very small voltages generated in the brain structures in response and time locked to specific events or stimuli such as sensory, motor or cognitive events
Reactivity	Eye opened /eye closed power ratio in each band

Table S6. PRISMA-DTA checklist

Section/topic	#	PRISMA-DTA Checklist Item	Reported on page #
**TITLE / ABSTRACT**			
Title	1	Identify the report as a systematic review (+/- meta-analysis) of diagnostic test accuracy (DTA) studies.	Page 1
Abstract	2	Abstract: See PRISMA-DTA for abstracts.	Page 1
**INTRODUCTION**			
Rationale	3	Describe the rationale for the review in the context of what is already known.	Page 2
Clinical role of index test	D1	State the scientific and clinical background, including the intended use and clinical role of the index test, and if applicable, the rationale for minimally acceptable test accuracy (or minimum difference in accuracy for comparative design).	Page 2
Objectives	4	Provide an explicit statement of question(s) being addressed in terms of participants, index test(s), and target condition(s).	Page 3
**METHODS**			
Protocol and registration	5	Indicate if a review protocol exists, if and where it can be accessed (e.g., Web address), and, if available, provide registration information including registration number.	NA
Eligibility criteria	6	Specify study characteristics (participants, setting, index test(s), reference standard(s), target condition(s), and study design) and report characteristics (e.g., years considered, language, publication status) used as criteria for eligibility, giving rationale.	Page 3
Information sources	7	Describe all information sources (e.g., databases with dates of coverage, contact with study authors to identify additional studies) in the search and date last searched.	Page 3
Search	8	Present full search strategies for all electronic databases and other sources searched, including any limits used, such that they could be repeated.	Supplemental Table S1
Study selection	9	State the process for selecting studies (i.e., screening, eligibility, included in systematic review, and, if applicable, included in the meta-analysis).	Page 3
Data collection process	10	Describe method of data extraction from reports (e.g., piloted forms, independently, in duplicate) and any processes for obtaining and confirming data from investigators.	Page 3, Supplemental file-2
Definitions for data extraction	11	Provide definitions used in data extraction and classifications of target condition(s), index test(s), reference standard(s) and other characteristics (e.g. study design, clinical setting).	Supplemental Table S5
Risk of bias and applicability	12	Describe methods used for assessing risk of bias in individual studies and concerns regarding the applicability to the review question.	Page 4
Diagnostic accuracy measures	13	State the principal diagnostic accuracy measure(s) reported (e.g. sensitivity, specificity) and state the unit of assessment (e.g. per-patient, per-lesion).	Page 4
Synthesis of results	14	Describe methods of handling data, combining results of studies and describing variability between studies. This could include, but is not limited to: a) handling of multiple definitions of target condition. b) handling of multiple thresholds of test positivity, c) handling multiple index test readers, d) handling of indeterminate test results, e) grouping and comparing tests, f) handling of different reference standards	NA-no metaanalysis

Section/topic	#	PRISMA-DTA Checklist Item	Reported on page #
Meta-analysis	D2	Report the statistical methods used for meta-analyses, if performed.	NA-no metaanalysis
Additional analyses	16	Describe methods of additional analyses (e.g., sensitivity or subgroup analyses, meta-regression), if done, indicating which were pre-specified.	NA-no metaanalysis
**RESULTS**			
Study selection	17	Provide numbers of studies screened, assessed for eligibility, included in the review (and included in meta-analysis, if applicable) with reasons for exclusions at each stage, ideally with a flow diagram.	Fig 1, page 5
Study characteristics	18	For each included study provide citations and present key characteristics including: a) participant characteristics (presentation, prior testing), b) clinical setting, c) study design, d) target condition definition, e) index test, f) reference standard, g) sample size, h) funding sources	Table 1, page 8
Risk of bias and applicability	19	Present evaluation of risk of bias and concerns regarding applicability for each study.	Supplemental Table S4
Results of individual studies	20	For each analysis in each study (e.g. unique combination of index test, reference standard, and positivity threshold) report 2x2 data (TP, FP, FN, TN) with estimates of diagnostic accuracy and confidence intervals, ideally with a forest or receiver operator characteristic (ROC) plot.	Table 4, page 16
Synthesis of results	21	Describe test accuracy, including variability; if meta-analysis was done, include results and confidence intervals.	Table 4, page 16
Additional analysis	23	Give results of additional analyses, if done (e.g., sensitivity or subgroup analyses, meta-regression; analysis of index test: failure rates, proportion of inconclusive results, adverse events).	NA
**DISCUSSION**			
Summary of evidence	24	Summarize the main findings including the strength of evidence.	Page 20-21
Limitations	25	Discuss limitations from included studies (e.g. risk of bias and concerns regarding applicability) and from the review process (e.g. incomplete retrieval of identified research).	Page 22-23
Conclusions	26	Provide a general interpretation of the results in the context of other evidence. Discuss implications for future research and clinical practice (e.g. the intended use and clinical role of the index test).	Page 23
**FUNDING**			
Funding	27	For the systematic review, describe the sources of funding and other support and the role of the funders.	Page 24

Adapted From: McInnes MDF, Moher D, Thombs BD, McGrath TA, Bossuyt PM, The PRISMA-DTA Group (2018). Preferred Reporting Items for a Systematic Review and Meta-analysis of Diagnostic Test Accuracy Studies: The PRISMA-DTA Statement. JAMA. 2018 Jan 23;319(4):388-396. doi: 10.1001/jama.2017.19163.

For more information, visit: **www.prisma-statement.org**.
