# Supplementary material for: The Role of EEG in the Diagnosis, Prognosis and Clinical Correlations of Dementia with Lewy Bodies—A Systematic Review
Source: Diagnostics (Basel). 2020 Aug 20;10(9):616. doi: 10.3390/diagnostics10090616 (PMC7555753; doi:10.3390/diagnostics10090616)
Supplement: Supplementary file 1 [file diagnostics-10-00616-s001.zip › Supplemental file-2 systematic review extraction forms Diagnostics.pdf]

Extraction form

Accession date:
Reviewer (abbreviation):

Title:

Author	Journal	Publication year	Center	Design

Inclusion criteria:

☐ Separate cohorts with Dementia with Lewy Bodies or Lewy Body Dementia
☐ Original research article
☐ Reported EEG findings
☐ Article in English
☐ With at least one of the objectives (tick)
- ☐ Differentiate LBD from other conditions
- ☐ Clinical correlate
- ☐ Assess EEG changes in relation to treatment
- ☐ Use EEG to prognosticate

Exclusion criteria:

☐ Local field potentials only

Patient population(s):

N=		Male:		Age (mean/sd)	
Groups:					
Disease duration (years; mean/sd):		Relation to DBS	Yes ☐<br>No ☐	Dementia:	Yes ☐<br>N.d. ☐<br>No ☐
Hoehn & Yahr stage:		Psychoactive medication:	Yes ☐<br>N.d. ☐<br>No ☐	Parkinsonian condition	On ☐<br>Off ☐<br>N.d. ☐

Aim:

EEG conditions:

Eyes-closed	☐
Resting-state	☐
Other conditions:	

EEG setup / analysis:

Number of electrodes		
# and duration of epochs		
Classical frequency domains	Yes ☐ No ☐	If no:
Bandpass filter		
Other:		

qEEG parameters studied:

Spectral analyses ☐	Connectivity ☐	Graph theory ☐
Specifically:		

Correlation qEEG parameters with:

Cognition ☐	Disease progression ☐	Motor function ☐
Psychiatric variables ☐	Treatment ☐	Other ☐
Specifically:		

Main conclusion(s):

Optional:

qEEG parameter	Outcome(s)
